# Development of a transcriptomic database for 14 species of scleractinian corals

**DOI:** 10.1186/s12864-019-5744-8

**Published:** 2019-05-17

**Authors:** Yanjie Zhang, Qian Chen, James Y. Xie, Yip Hung Yeung, Baohua Xiao, Baoling Liao, Jianliang Xu, Jian-Wen Qiu

**Affiliations:** 1 HKBU Institute of Research and Continuing Education, Virtual University Park, Gaoxin South 4th Road, Shenzhen, 518057 China; 2 Department of Biology, Hong Kong Baptist University, Hong Kong, China; 3 Department of Computer Science, Hong Kong Baptist University, Hong Kong, China; 4 Present address: Google China, Beijing, China; 5 Shenzhen Institute of Guangdong Ocean University, Shenzhen, China

**Keywords:** Coral, Coral reef, Database, Scleractinia, Symbiotic algae, Transcriptome

## Abstract

**Background:**

Scleractinian corals are important reef builders, but around the world they are under the threat of global climate change as well as local stressors. Molecular resources are critical for understanding a species’ stress responses and resilience to the changing environment, but such resources are unavailable for most scleractinian corals, especially those distributed in the South China Sea. We therefore aimed to provide transcriptome resources for 14 common species, including a few structure forming species, in the South China Sea.

**Description:**

We sequenced the transcriptome of 14 species of scleractinian corals using high-throughput RNA-seq and conducted de novo assembly. For each species, we produced 7.4 to 12.0 gigabases of reads, and assembled them into 271 to 762 thousand contigs with a N50 value of 629 to 1427 bp. These contigs included 66 to 114 thousand unigenes with a predicted open reading frame, and 74.3 to 80.5% of the unigenes were functionally annotated. In the azooxanthelate species *Tubastraea coccinea*, 41.5% of the unigenes had at least a best-hit sequence from corals. In the other thirteen species, 20.2 to 48.9% of the annotated unigenes had best-hit sequences from corals, and 28.3 to 51.6% from symbiotic algae belonging to the family Symbiodinaceae*.* With these resources, we developed a transcriptome database (CoralTBase) which features online BLAST and keyword search for unigenes/functional terms through a user friendly Internet interface.

**Short conclusion:**

We developed comprehensive transcriptome resources for 14 species of scleractinian corals and constructed a publicly accessible database (www.comp.hkbu.edu.hk/~db/CoralTBase). CoralTBase will facilitate not only functional studies using these corals to understand the molecular basis of stress responses and adaptation, but also comparative transcriptomic studies with other species of corals and more distantly related cnidarians.

**Electronic supplementary material:**

The online version of this article (10.1186/s12864-019-5744-8) contains supplementary material, which is available to authorized users.

## Background

Coral reefs are ecologically and economically important, but around the world they are threatened by global climate change such as ocean warming and acidification [1, 2], as well as local stressors such as poor fishing practices, pollution, coastal development, and unsustainable recreational activities [3–5]. Over the last several decades, coral reefs in many regions have degraded dramatically [6, 7]. A comprehensive assessment of 704 species of reef-building corals around the world placed 231 species (32.8%) in categories with elevated risk of extinction [8]. In Southeast Asia, around 50% of coral reefs are facing high or very high threat of degradation [9]. Along the northern coasts of the South China Sea, dramatic reduction in live coral cover and changes in dominant coral species have occurred over the last several decades in Hainan [10] and Guangdong [11] provinces.

Scleractinia, commonly called hard corals or stony corals due to their calcified skeleton, are often important reef builders. Around the world there are 1605 extant scleractinian species, which are classified into 304 genera and 40 families [12]. In recent years, it has been increasingly realized that developing molecular resources, especially transcriptome and genome sequences, can facilitate studies aiming to understand mechanisms underlying coral stress responses and resilience in the changing environment [13, 14]. Nevertheless, our survey in January 2019 showed that only a small fraction of scleractinian species (i.e. 35 species representing 20 genera and 11 families) have transcriptome data deposited in the National Center for Biotechnology Information (NCBI) database and Reefgenomics (Additional file 1:Table S1). An analysis of the datasets with collection site information shows that the geographic distribution of such transcriptomic resources is biased: 6, 9, and 9 of the transcriptomes were produced based on samples collected from the Great Barrier Reef, the Caribbean Sea, and East Asia, respectively. Only 5 were based on species distributed in the South China Sea, which in total hosts 571 species of scleractinians [15]. In addition, there were reports showing genetic differentiation among coral populations in different regions [16–19], therefore it is valuable to develop population-specific transcriptomes.

We therefore aimed to provide comprehensive transcriptomic resources for a set of common scleractinian corals in the South China Sea. Based on samples collected from Hong Kong, we sequenced and assembled the transcriptomes for 14 species of scleractinians representing 8 families and 14 genera: Fungiidae (*Lithophyllon undulatum*), Faviidae (*Leptastrea purpurea*), Merulinidae (*Favites acuticollis*, *Platygyra carnosa*, *Hydnaphora exesa*, *Dipsastraea rotuman*), Acroporidae (*Montipora peltiformis*, *Acropora digitifera*), Euphylliidae (*Galaxea fascicularis*), Agariciidae (*Pavona decussata*), Poritidae (*Goniopora lobata*, *Porites lutea*), Dendrophylliidae (*Turbinaria peltata*, *Tubastraea coccinea*). These species covered the most common species of scleractinian corals in Hong Kong, including several species (i.e. *A. digitifera*, *P. carnosa*, *M. peltiformis* and *P. decussata*) that are important in forming reef structures [20]. Although a transcriptome of *P. carnosa* from Hong Kong is already available [21], its completeness is quite low, with only 73.42% complete BUSCOs (Benchmarking Universal Single-Copy Orthologs). In recent years, the health of some of these coral species has been affected by various stressors including excessive bioerosion [22–24], skeletal growth anomalies [25], bleaching [26], and recreational activities [27, 28]. To facilitate easy access to the transcriptome data, we constructed a relational database with a user-friendly Internet interface.

## Construction and content

### Collection of coral samples

**Fig. 1:**
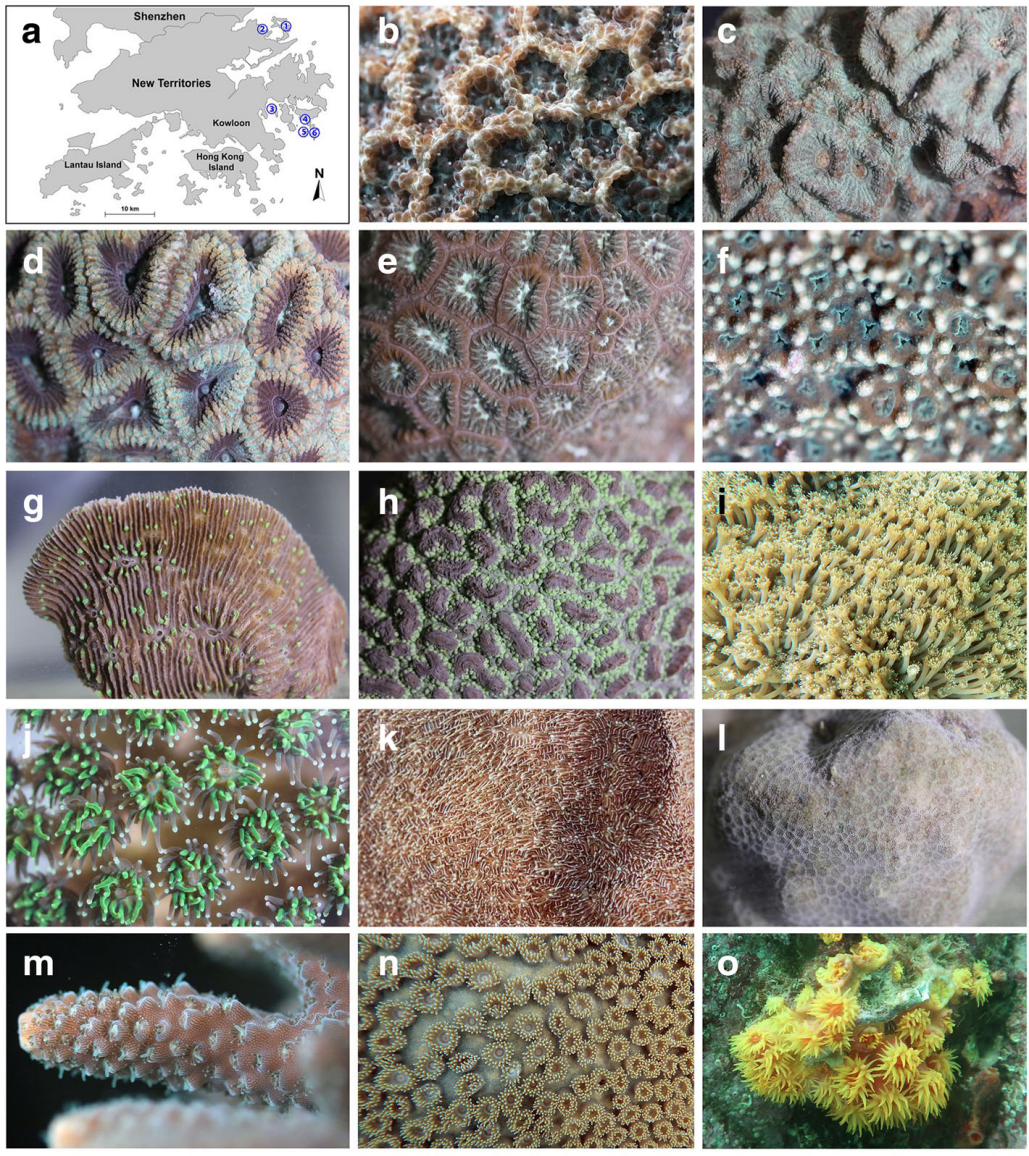
Corals included in the database construction. **a**, Map of Hong Kong showing the coral collection sites: Crescent Island (①); Lai Chi Wo (②); Bluff Island (③); Pak A (④); Sharp Island North (⑤); Basalt Island (⑥). **b**-**o** Photographs showing the external morphology of the coral polyps: *Platygyra carnosa* (**b**) *Favites acuticollis* (**c**) *Dipsastraea rotumana* (**d**) *Leptastrea purpurea* (**e**) *Montipora peltiformis* (**f**) *Lithophyllon undulatum* (**g**) *Hydnophora exesa* (**h**) *Goniopora lobate* (**i**) *Galaxea fascicularis* (**j**) *Pavona decussata* (**k**) *Porites lutea* (**l**) *Acropora digitifera* (**m**) *Turbinaria peltata* (**n**) *Tubastraea coccinea* (**o**)

The following 14 species of stony corals were collected from six sites in Hong Kong from June to July 2017 by SCUBA diving (Fig. 1): *P. decussata* from Sharp Island North; *G. lobata*, *P. lutea*, *L. undulatum*, *L. purpurea* and *G. fascicularis* from Crescent Island; *A. digitifera*, *T. peltata*, *M. peltiformis*, *D. rotumana* and *F. acuticollis* from Bluff Island; *H. exesa* from Pak A; *T. coccinea* from Basalt Island; and *P. carnosa* from Lai Chi Wo. For each species, three small colonies (~ 2 cm^2^) were collected, put in a cooler with dry ice immediately once they were brought out of the sea surface, transported to Hong Kong Baptist University where they were stored in a freezer at − 80 °C until use.

### RNA extraction and RNA-seq

Total RNA was extracted from each sample using TRIzol reagent (Invitrogen, Carlsbad, CA, USA) following the manufacturer’s protocol. After treatment with RNase-free DNase I (ThermoFisher Scientific, Waltham, MA, USA), the quality of RNA samples was determined using 1% agarose gel electrophoresis and the quantity was determined using a NanoDrop 2000c Spectrophotometer (ThermoFisher Scientific, Waltham, MA, USA). RNA samples from three colonies for each species were pooled, then sent to Beijing Genomics Institute (BGI), Shenzhen for transcriptomic sequencing using an Illumina X-TEN platform. Before the library preparation, the concentration of the RNA samples was further analyzed using a Bioanalyzer 2100 (Agilent Technologies, CA, USA). Oligo dT enrichment was used during the library construction with a NEBNext Poly(A) mRNA Magnetic Isolation Module kit (New England Biolabs, MA, USA). The library was prepared using a NEBNext Ultra RNA Library Prep Kit for Illumina (New England Biolabs, MA, USA). Sequencing was conducted under the paired-end mode to produce reads 151 bp in length. All sequences were cleaned to remove adaptors and low-quality reads with a high proportion of N (> 10%) or high proportion of low-quality (Phred value Q ≤ 20) nucleotide base (> 40%). The clean reads are deposited in the Sequence Read Archive (SRA) of NCBI under accession number PRJNA512264.

### Transcriptome assembly, completeness assessment, and annotation

Clean reads of each species were assembled using Trinity 2.5.1 [29] under the default settings. Transcript abundance was estimated as transcripts per kilobase million read (TPM) using RSEM 1.2.19 [30], and those without expression or very low expression (TPM < 0.5) were removed manually. Candidate open reading frames (ORFs) and peptides were identified from the transcripts using TransDecoder, and duplicate sequences with 100% similarity in predicted peptides were removed using CD-HIT [31]. For each species, the completeness of the assembled transcriptome was assessed using BUSCO (benchmarking universal single-copy orthologs) v1.1b [32] with a set of 978 conserved single-copy metazoan genes as the reference. Unigenes (i.e. the longest isoform for each gene) were annotated using both Diamond v0.9.19.120 [33] and InterProScan-5.13-52.0 [34]. Specifically, general sequence annotation was conducted using Diamond v0.9.19.120, which applied BLASTp search against NCBI’s non-redundant (nr) database with an E-value of 1 × 10^− 5^. To determine the protein domain structure and its functional features, Gene Ontology (GO) function, Kyoto Encyclopedia of Genes and Genomes (KEGG) and Reactome pathways for each unigene were classified using InterProScan-5.13-52.0 under default settings.

For each of the 14 species, RNA-seq produced 7.4 to 12 Gb clean reads (Table 1). Transcriptome assembly produced 271,569 to 762,693 contigs with an N50 of 629 to 1610. These contigs contained 259,788 to 495,155 predicted proteins. After removing unigenes with low expression level (TPM < 0.5) and the identical sequences, there were 66,342 to 113,634 unigenes left in the sequenced stony corals for use in downstream analyses.

**Table 1:** Summary of transcriptome assembly results for 14 species of corals

Species	*G. lobata*	*P. lutea*	*L. undulatum*	*L. purpurea*	*G. fascicularis*	*A. digitifera*	*T. peltata*	*M. peltiformis*	*D. rotumana*	*F. acuticollis*	*H. exesa*	*T. coccinea*	*P. decussata*	*P. carnosa*
Sequences														
No. clean reads (× 10^6^)	53.5	52.9	57.3	54.6	57.2	56.8	57.1	58.0	57.4	57.1	52.1	80.1	49.6	71.6
No. clean bases (× 10^6^)	8020.5	7931.8	8592.8	8190.4	8575.9	8525.0	8567.1	8692.7	8608.4	8570.6	7814.9	12,011.7	7446.6	10,703.7
Q20 (%)	94.43%	95.03%	94.69%	95.02%	95.09%	94.78%	94.96%	95.17%	95.22%	95.25%	94.86%	97.20%	94.55%	97.78%
GC (%)	48.59%	46.62%	47.16%	48.40%	45.46%	47.21%	49.68%	44.41%	47.00%	44.99%	47.06%	44.82%	48.36%	48.68%
Assembly														
Contigs	343,691	361,384	329,100	612,181	324,237	271,569	563,805	314,269	583,587	389,239	677,268	529,492	762,693	329,657
GC%	46	45	45	46	45	45	46	45	47	45	46	44	44	47
N50 (bp)	1252	1181	1427	979	1473	1610	1072	1292	629	1337	946	1053	1077	1132
No. peptides	298,424	277,066	293,531	468,029	289,072	281,219	435,029	259,788	364,047	306,486	495,155	316,983	452,102	274,164
No. unigenes	78,532	74,781	70,872	114,375	70,841	67,587	98,266	66,561	93,470	75,250	107,504	70,018	86,616	107,565
Retained unigenes	78,193	74,484	70,555	113,634	70,576	67,367	97,609	66,342	93,004	74,923	106,640	69,464	85,210	104,573
Completeness assessment														
Complete (%)	895 (91.51%)	907 (92.74%)	898 (91.82%)	911 (93.15%)	895 (91.51%)	874 (89.37%)	880 (89.98%)	897 (91.72%)	842 (86.09%)	899 (91.92%)	906 (92.64%)	925 (94.58%)	869 (88.85%)	893 (91.31%)
Partial (%)	40 (4.09%)	30 (3.07%)	33 (3.37%)	44 (4.50%)	30 (3.07%)	27 (2.76%)	57 (5.83%)	22 (2.25%)	88 (9.00%)	38 (3.89%)	47 (4.80%)	36 (3.68%)	54 (5.53%)	49 (5.01%)
Missing (%)	43 (4.40%)	41 (4.19%)	47 (4.81%)	23 (2.35%)	53 (5.42%)	77 (7.87%)	41 (4.19%)	59 (6.03%)	48 (4.91%)	41 (4.19%)	25 (2.56%)	17 (1.74%)	55 (5.62%)	36 (3.68%)
Unigene annotation														
Total number	61,440	58,120	56,529	86,253	54,862	54,249	73,723	51,685	71,551	57,922	79,394	53,035	63,291	78,309
Annotated by nr database	58,935	55,932	54,422	82,138	52,919	52,195	69,753	49,930	68,939	55,943	75,512	50,544	60,674	74,528
Annotated by Interproscan	39,253	36,023	35,383	58,646	33,451	33,469	50,410	31,360	42,071	36,407	55,404	39,467	39,111	46,109
With GO	31,308	28,816	28,188	47,479	26,694	26,827	40,852	25,142	33,059	29,274	45,268	32,664	30,913	35,866
With KEGG & REACTOME	7899	6664	6222	12,720	6040	5935	10,877	5888	8339	7217	13,102	9157	6608	7700
No. coral genes	19,610	22,621	22,673	20,913	21,254	19,806	16,253	20,569	27,993	22,226	15,287	21,993	29,689	31,327
No. algal genes	26,022	26,197	26,019	24,784	25,861	26,939	23,829	23,888	30,427	23,861	21,415	154	26,056	35,225

The transcriptomes were assessed for the presence of the 978 core metazoan BUSCOs, which showed that they contained 86.09 to 94.58% complete BUSCOs, and 2.76–9.00% partial BUSCOs (Table 1). These metrics are comparable with those of recently published coral transcriptomes [35, 36], indicating the high completeness of our transcriptome assemblies.

### Proportion of sequences from coral and symbiotic algae

Unigenes from each species were annotated by BLAST search against NCBI nr database and InterProscan. For each species, 51,685 to 86,253 unigenes were successfully annotated, which accounted for 74.3 to 80.5% of the total unigenes (Table 1). Consistent with the expectation that members of the genus *Tubastraea* are azooxanthellate, 43.5% of the annotated *T. coccinea* unigenes had best hits from corals; only 0.3% of the annotated unigenes had best hit sequences from *Cladocopium* (formerly *Symbiodinium* clade C [37]), which likely came from the environmental water or reef inhabitants that had symbiotic algae. Among the annotated unigenes from the 13 zooxanthellate species, 20.2 to 48.9% unigenes had best-hit sequences from corals, and 28.3 to 51.6% from symbiotic algae*.* Among the unigenes, 45.8 to 61.6% were successfully annotated with GO terms, and 9.8 to 17.3% with KEGG and Reactome.

### The identities of symbiotic algae

To determine the identities of symbiotic algae in the corals, we searched our coral transcriptome data for several gene fragments in two ways. First, we conducted local BLAST against the GeoSymbio database [38] to search for *ITS2* genes, after adding the *ITS2* Symbiodiniaceae sequences reported from several species of corals in Hong Kong [39]. Our query returned subclade C1 as the best hit sequence in 10 of 13 sequenced corals that have symbionts (i.e. *G. lobata*, *P. lutea*, *L. undulatum*, *L. purpurea*, *A. digitifera*, *T. peltata*, *F. acuticollis*, *H. exesa*, *P. decussata*, *P. carnosa*) (Additional file 1: Table S2A). Subclade C15 was the best hit for *Porites lutea*. However, there was no *ITS2* BLAST result for the symbionts of *G. fascicularis*, *M. peltiformis* and *D. rotumana*, probably because the Oligo dT enrichment procedure used in the library construction had removed all of the ribosomal RNA sequences including *ITS2* in these three species.

Second, we conducted local BLAST against several Symbiodiniaceae markers (chloroplast *23S* rRNA genes, *18S* rRNA, *ITS1*, *5.8S* rRNA and *28S* rRNA) that have been used to identify symbiotic algal types. The accession numbers of sequences of these other markers used in local BLAST are listed in Additional file 1: Table S3. To improve the accuracy of the BLAST results, the e-value threshold was set as 1e × 10^− 100^ and the identity larger than 98%. Our query returned *Symbiodinium* clade C (i.e. *Cladocopium* [37]) as the best-hit taxon for most of our transcriptomes, with some annotations also contained the subclade information (Additional file 1: Table S2B). Specifically, for the three species whose symbiont type could not be identified based on *ITS2*, both subclade C1 and C3 were the best hit for *G. fascicularis* and *D. rotumana* (based on 5.8S rRNA, ITS2, 28S rRNA and chloroplast 23S rRNA) and subclade C1 for *M. peltiformis* (based on 5.8S rRNA, ITS2, 28S rRNA). For the azooxanthellate coral *Tubastraea coccinea*, BLAST returned only one sequence from Symbiodiniaceae but its very low expression level (TMP = 0.56) indicated that the sequences were contaminants from the environment.

### Database structure

CoralTBase, a relational database, was constructed using a method described previously [21, 40] to provide access to the 14 assembled coral transcriptomes through the Internet. Users can search data from one species or multiple species at the same time. The database, constructed using MySQL v5.6.34, is hosted on an Apache HTTP server. The data include DNA and protein sequences of all unigenes, which are linked with their corresponding NCBI nr, GO and KEGG and Reactome annotations by unigene ID. The database contains two relation tables (“GO_relation” and “KEGG_and_Reactome_relation”) and five entity tables (“NCBI annotation”, “Proteins”, “DNAs”, “GO” and “KEGG and Reactome”). A stand-alone web server, powered by ViroBLAST [41], was incorporated in the database to allow for BLAST search.

## Utility and discussion

### Layout of CoralTBase

**Fig. 2:**
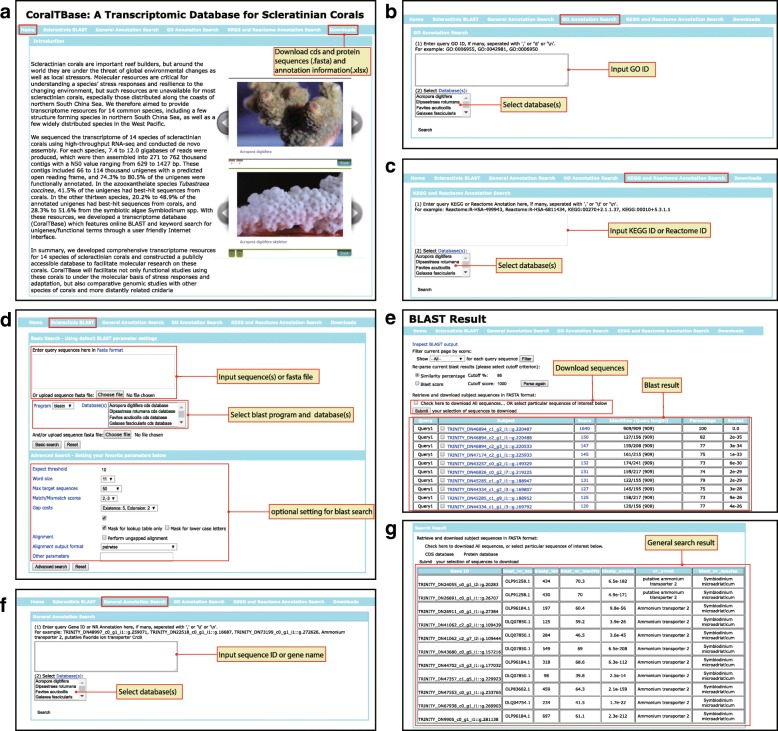
The web interface of CoralTBase. **a** The front page. **b** Illustration of query by GO annotation. **c** Illustration of query by KEGG and Reactome annotation. **d** Illustration of the Basic and Advanced BLAST search options. **e** An example of the search result of a BLAST search, showing matched sequences, each with their BLAST statistics. **f** Illustration of the general annotation search. **g** An example of the search result of general annotation search

CoralTBase can be accessed at www.comp.hkbu.edu.hk/~db/CoralTBase. Users can search the data from one or multiple species in several ways by BLAST or by a number of other query terms (Fig. 2). BLAST supports queries using DNA/protein sequence or fasta-format file against NCBI nr database (Fig. 2d). The output is a list of gene or protein sequences that match the query sequence with an E-value and similarity score (Fig. 2e). The returned DNA or protein sequence contains an attribute “Unigene ID” as well as its corresponding annotation. General Annotation Search allows users to query gene annotation (i.e. NCBI annotation) by gene name (e.g. ammonium transporter 2, Fig. 2f and g) or sequence ID. GO Annotation Search is the search method according to the GO class ID (Fig. 2b). A successful search will return a table that contains the matched Go class ID, and the unigene ID. KEGG and Reactome Annotation Search will return a table containing the KEGG or Reactome pathway and the matched unigenes (Fig. 2c). The DNA and protein sequences of all unigenes for each species can be downloaded from the Downloads area.

We used the host genes in the transcriptome of *A. digitifera* as an example to show the potential utility of the resource. We prepared a figure showing the GO annotations of the host genes (Additional file 3: Figure S1a). For the same species, we also plot the Wnt pathway (Additional file 3: Figure S1b). The Wnt pathway plays important roles in biomineralization and osteogenesis in vertebrates [42, 43] and has been reported in the transcriptome of the stony coral *Stylophora pistillata* [44]. We found that all Wnt genes in the KEGG pathway for *A. digitifera* can be found in our transcriptome obtained in this study. Moreover, we found a few more genes (in red boxes) in the Wnt signaling pathway from our transcriptome, which is currently not present in the KEGG networks for *A. digitifera*. This example indicates that the transcriptome obtained in this study has a high coverage and it will be useful for further analysis of coral biology.

**Fig. 3:**
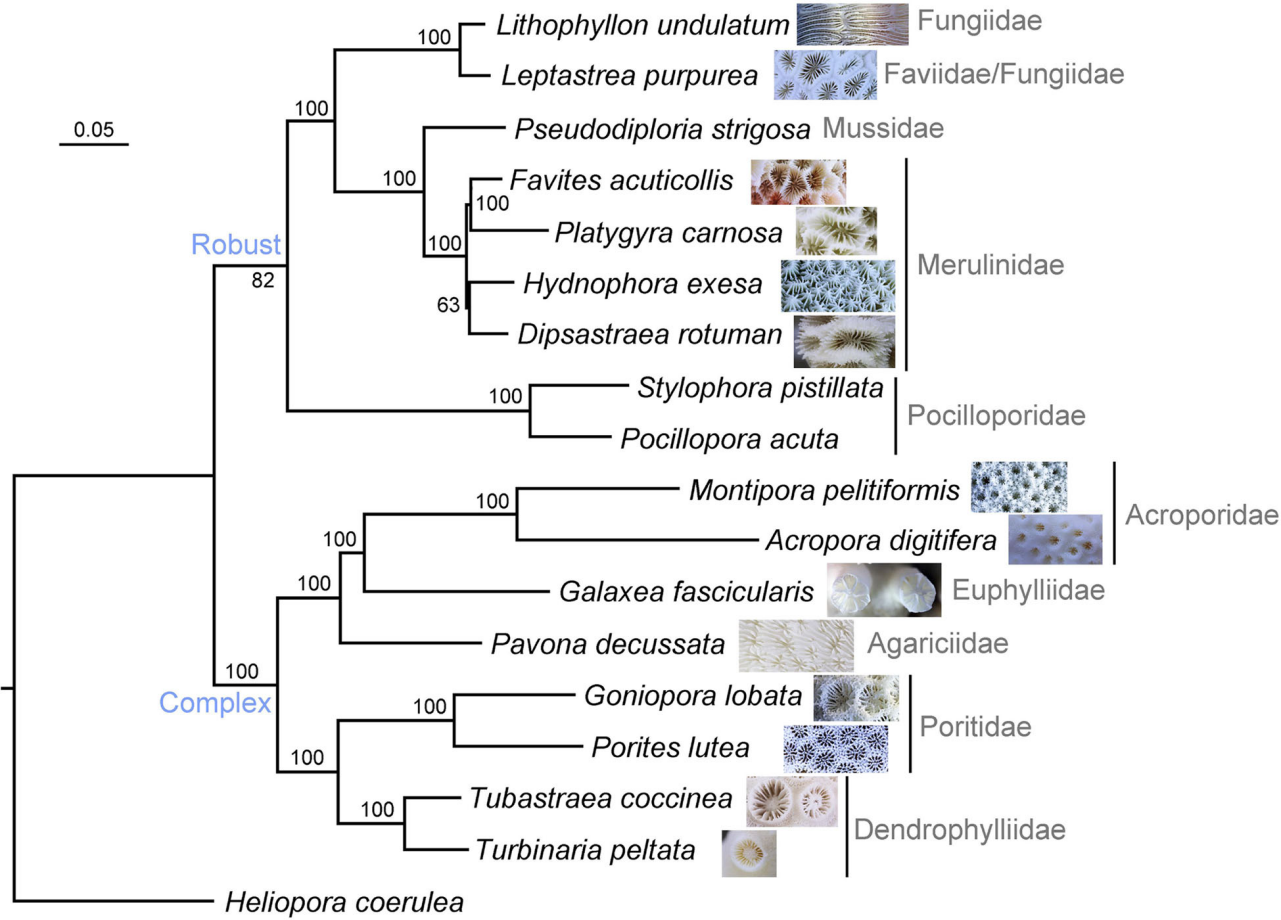
Phylogenetic tree of Scleractinia constructed based on one-to-one orthologous genes of 18 species. An image of the skeleton of each coral species is shown on the right of the species name. Numbers on main branches are bootstrap values in ML analysis. The transcriptomes of the stony coral *Pocillopora damicornis*, *Pseudodiploria strigosa*, *Stylophora pistillata* were downloaded from NCBI GenBank TSA database. *Heliopora coerulea* belongs to the order Helioporacea was used as the outgroup

We obtained 132 one-to-one homologous genes from 18 species including all species we sequenced as well as four species whose data were downloaded from the GenBank. Based on these homologous genes we constructed a phylogenetic tree to show their evolutionary relationships (Fig. 3), using a method detailed in Additional file 3: Methods. We also provided the sequences alignment in Additional file 2: Alignment.

### Potential applications and expansion

The resources produced in this study can be used to understand basic coral biology such as stress responses, development, reproduction, symbiosis and calcification. They can also be used as a transcriptomic reference for Tag-seq, which is more cost-effective and accurate traditional RNA-seq at quantifying gene expression [45]. Such studies can be conducted to understand the molecular mechanisms underlying various responses to stressors, such as high temperature, low salinity and disease development [46–48]. In a broader taxonomic context, these resources can be used in comparative genomic studies aiming to understand the evolution of early development [49], biomineralization [50], and immunity [51]. In the future, CoralTBase can be expanded to include more scleractinian and non-scleractinian species. For the species that have been included in the database, the transcriptome can be updated with data from more developmental stages or from different populations.

## Conclusions

This work has generated high-throughput transcriptome data for 14 species of scleractinian corals. It has increased the number of scleractinian corals around the world with transcriptome dataset from 35 species to 45 species, 20 genera to 26 genera and 11 families to 13 families. For some species with published transcriptome database already, our new data are either more comprehensive (i.e. *Platygyra carnosa*) or are based on specimens collected from different geographical areas and therefore represent different populations (i.e. *A. digitifera, G. fascicularis and P. lutea*). We have also organized the transcriptome data into a relational database to facilitate easy access by the public.

## Additional files

Additional file 1: **Table S1.** Information on published transcriptome datasets from Scleractinia. **Table S2.** Symbiotic algae types determined by BLAST coral transcriptomes against the ITS2 and rRNA genes (i.e. 18S, 28S 23S rRNA) from GenBank database. **Table S3.** The accession numbers of sequences in the GenBank database used for symbiotic algae clade identification. (XLSX 179 kb)

Additional file 2: Alignment. The alignment of one-to-one homologous genes of 18 stony coral species. (FASTA 3031 kb)

Additional file 3: Method. Method for phylogeny of Scleractinia and **Figure S1.** (DOCX 361 kb)

## Abbreviations

BGI: Beijing Genomics Institute
GO: Gene ontology
ITS: Internal transcribed spacer
KEGG: Kyoto Encyclopedia of Genes and Genomes
ML: Maximum likelihood
nr database: non-redundant database
ORFs: Open reading frames
rRNA: ribosomal RNA
TPM: Transcripts per million
