# Supplementary material for: Development of a transcriptomic database for 14 species of scleractinian corals
Source: BMC Genomics. 2019 May 17;20:387. doi: 10.1186/s12864-019-5744-8 (PMC6525400; doi:10.1186/s12864-019-5744-8)
Supplement: Supplementary file 3 — Method. Method for phylogeny of Scleractinia and Figure S1. (DOCX 361 kb) [file 12864_2019_5744_MOESM3_ESM.docx]

**Additional file**

***Alignment of homologous genes and phylogeny of Scleractinia***

Eighteen species were used to illustrate the evolutionary relationship of stony corals. The transcriptomes of three stony corals were downloaded from NCBI GenBank TSA database: accession number GEFF00000000.1 for *Pocillopora damicornis*, HACD00000000.1 for *Pseudodiploria strigosa*, and GARY00000000.1 for *Stylophora pistillata*. *Heliopora coerulea* belonging to the order Helioporacea was used as the outgroup. Its assembled transcriptome was downloaded from NCBI GenBank TSA dataset (accession number IABP00000000.1). OrthoMCL (Li et al., 2003) was used to identify the ortholog groups. One-to-one orthologs were used for the phylogenetic analysis. Gene alignment was done using ClustalW in ParaAT (Zhang et al., 2012). The aligned genes were concatenated manually. The poorly aligned regions were removed using gblocks v. 0.91 b (Castresana, 2002). Phylogenetic analysis was conducted using the maximum likelihood (ML) method. The ML analysis was conducted using RaXML GUI 1.3 (Silvestro & Michalak, 2012) with the GTR +I+G model which was determined using jModelTest-2.1.1 (Darriba et al., 2012). The bootstrap values were based on 1000 iterations. In total, 132 one-to-one orthologs (57,957 sites) were used to conduct the phylogenetic tree.

**Additional file Figure S1**


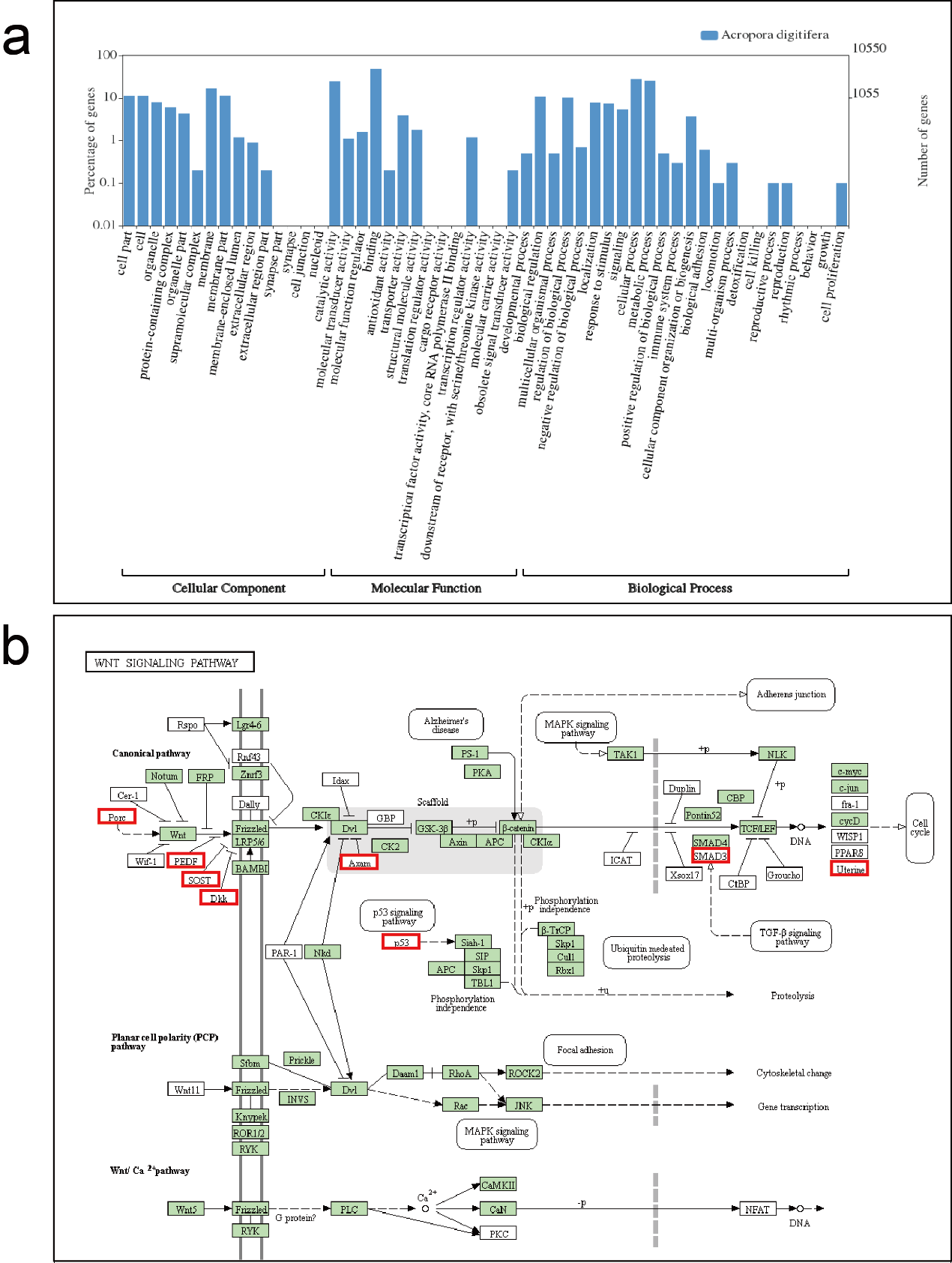


**Additional figure S1.** Examples illustrating the use of GO annotation and KEGG pathway for *Acropora digitifera*. **(a)** Gene Ontology Annotation Plot, **(b)** Wnt signaling pathway downloaded from the KEGG Pathway database. Genes in a green box are found in *A. digitifera* from both the KEGG database and our transcriptome dataset. Genes framed in a red box are the additional genes found only in our transcriptome of *A. digitifera*.

**Reference**

Castresana, J. (2002). Gblocks, v. 0.91 b. *Online version available at: http://molevol. cmima.csic.es/castresana.Gblocks_server.html*.

Darriba, D., Taboada, G. L., Doallo, R., & Posada, D. (2012). jModelTest 2: more models, new heuristics and parallel computing. *Nature Methods*, *9*(8), 772.

Li, L., Stoeckert, C. J., & Roos, D. S. (2003). OrthoMCL: identification of ortholog groups for eukaryotic genomes. *Genome Research*, 13(9), 2178-2189.

Silvestro, D., & Michalak, I. (2012). raxmlGUI: a graphical front-end for RAxML. *Organisms Diversity & Evolution*, *12*(4), 335-337.

Zhang, Z., Xiao, J., Wu, J., Zhang, H., Liu, G., Wang, X., & Dai, L. (2012). ParaAT: a parallel tool for constructing multiple protein-coding DNA alignments. *Biochemical and Biophysical Research Communications*, 419(4), 779-781.
